# A randomized clinical trial on the effect of foot reflexology performed in the fourth stage of labor on uterine afterpain 

**DOI:** 10.1186/s12884-022-04376-w

**Published:** 2022-01-21

**Authors:** Neda Sharifi, Narjes Bahri, Fatemeh Hadizadeh-Talasaz, Hoda Azizi, Hossein Nezami, Hamid Reza Tohidinik

**Affiliations:** 1 Department of Midwifery, Faculty of Nursing and Midwifery,Mashhad Medical Sciences, Islamic Azad University, Mashhad, Iran; 2 Department of Midwifery, Faculty of Medicine, Social Determinants of Health Research Center, Gonabad University of Medical Sciences, Gonabad, Iran; 3 Department of Midwifery, Faculty of Medicine, Social Development and Health Promotion Research Center, Gonabad University of Medical Sciences, Gonabad, Iran; 4 Department of Chinese and Complementary Medicine, School of Persian and Complementary Medicine, Mashhad University of Medical Sciences, Mashhad, Iran; 5 Department of Epidemiology and Biostatistics, School of Health, Mashhad University of Medical Sciences, Mashhad, Iran; 6 Centre for Academic Child Health, Population Health Sciences, Bristol Medical School, University of Bristol, Bristol, UK; 7 Department of Biostatistics and Epidemiology, School of Public Health, Kerman University of Medical Sciences, Kerman, Iran

**Keywords:** Reflexology, Afterpain, Complementary medicine

## Abstract

**Background:**

Uterine afterpains are among the most prevalent complaints after natural vaginal delivery. Non-pharmacological treatment modalities to relieve afterpains are an important care priority. This study was conducted to investigate the effect of foot reflexology in the fourth stage of labor on afterpains in multiparous women.

**Methods:**

This single-blind, randomized, clinical trial recruited 80 eligible pregnant women presenting to Allameh Bohlool Hospital in Gonabad and 17-Shahrivar Hospital in Mashhad, Iran, in 2019. In the first and second hours postpartum, the reflexology group received 10 min of general massage and specific reflexology massage on each foot on the uterine, pituitary, and solar plexus points. The control group received 10 min of general massage, and then rotational massage on a neutral point on the lateral side of the heel. The pain was measured every hour up to 4 h postpartum using a visual analogue  scale. The collected data were analyzed in SPSS-16 software at a significance level of *P* < 0.05.

**Results:**

The median of the afterpain score in the first hour (*P* = 0.05) and second hour (*P* = 0.274) postpartum did not differ significantly between the two groups, but this score was lower in the reflexology group at the third hour (*P* < 0.001) and fourth hour (P < 0.001) postpartum. The mean total afterpain score in the 4 h postpartum was significantly lower in the reflexology group (P < 0.001).

**Conclusion:**

The results revealed that foot reflexology in the fourth stage of labor has positive effects on relief from uterine afterpain. Reflexology is therefore recommended as a measure to reduce postpartum uterine afterpains.

## Background

Pregnancy, labor, and puerperium are associated with many changes in women’s body and can affect their health [1]. Uterine afterpains are reportedly the most prevalent postpartum pain (77%) in multiparous women [2]. Uterine afterpains usually last two to 4 days postpartum and resolve spontaneously. Factors affecting pain intensity include multiparity, uterine overdistention (multiple pregnancies, large fetus, polyhydramnios), breastfeeding, prolonged labor stages, analgesia during labor, maternal physical and mental disorders, history of dysmenorrhea, maternal weight, and cultural factors such as race, education and religion [3–11]. The severity of afterpains increases in multiparous women due to the increased sensitivity of their central nervous system and decreased uterine muscle tone following multiple deliveries or uterine overextension, while nulliparous women experience little or no afterpain because their uterus has high muscle tone and contracts well [3, 4, 12].

Afterpains can lead to maternal and neonatal complications including anxiety, sleep disturbances, emotional disorders, depression, inability to care for the baby and continue breastfeeding, delays in early breastfeeding, and reduced maternal ability to perform daily chores. James et al. (2008) reported pain in the early hours postpartum as one of the most important factors contributing to chronic postpartum pain and depression [13]. Furthermore, pain and stress increase adrenaline release and decrease oxytocin release, thus leading to the cessation of the oxytocin reflex and disrupting breast milk production in addition to causing discomfort for the mother [14].

The most common method used to relieve afterpains is the administration of oral analgesics such as acetaminophen and ibuprofen [15]. Nonetheless, some analgesics (e.g., mefenamic acid and ibuprofen) occasionally cause side effects such as nausea, vomiting, diarrhea, abdominal pain, bleeding, gastrointestinal obstruction or perforation, dizziness, drowsiness, seizures, acute renal failure, and interstitial nephritis [16, 17]. In the United States, the serious side effects of nonsteroidal anti-inflammatory drugs (NSAIDs) have resulted in 100,000 hospitalizations and more than 16,500 deaths [17]. Medication side effects have drawn attention to non-pharmacological pain management modalities, including reflexology. During breastfeeding, women are very much concerned about their drug intake, since some amount of any drug they take (about 1 %) enters the breast milk [18].

Reflexology is a branch of complementary medicine and an old and non-invasive method that involves massaging reflex points on the hand and legs. This method can reduce cortisol and adrenaline levels, increase the secretion of serotonin, endorphins and enkephalins, increase vasodilation in the peripheral arteries and improve blood flow, eliminate toxins, and boost the immune system by acting on the interstitial fluid and connective tissue throughout the body [19]. Reflexology generates electrochemical messages and thus stimulates certain nerve points, and this action is done to reduce stress and balance the body [20].

A systematic review study also showed that reflexology during different stages of labor reduces labor pain severity [21].

Although numerous studies have reported the effect of reflexology on labor pain and post-cesarean section pain, an extensive review of literature up to the time of writing this paper yielded no studies regarding the effects of reflexology on the other stages of labor, including the fourth stage and the pain experienced after natural delivery. Since reflexology is a non-invasive modality that can be used along conventional therapies to reduce afterpains and mitigate the side effects of chemical medications, the present study was conducted to investigate the effect of foot reflexology performed in the fourth stage of labor on afterpains.

## Methods

### Trial design

The protocol of this randomized clinical trial was approved by the Ethics Committee of Gonabad University of Medical Sciences (IR.GMU.REC.1397.079). The study was also registered at the Iranian Registry of Clinical Trials on 4 February 2019 (IRCT20181214041962N1). The report of this clinical trial is based on the CONSORT 2010 checklist [22].

### Participants and setting

Pregnant women referring to Allameh Bohlool Gonabadi Hospital in Gonabad and 17-Shahrivar Hospital in Mashhad, Iran, from February 2019 to July 2019, were enrolled for this study. The most important inclusion criteria were: Having no history of postpartum hemorrhage, gravida 2 or 3, gestational age of 37–42 weeks, no high-risk pregnancy, and no uterine overdistention. The exclusion criteria were: Unwillingness of the mother to continue participation, manual removal of the placenta and membranes, the need for additional treatments to control bleeding, and neonatal birth weight over 4 kg. The inclusion and exclusion criteria are fully described in the paper that reports the study protocol [23].

### Sample size and randomization

The results reported by Yousefi et al. (2011) were used for determining the sample size required for comparing the two groups in G*Power software, version 3.1.9.2 [24], and t-tests were used for this purpose. In this study, the effect size d was 0.712, type I error was a maximum of 5%, and the test power was 80%, making the overall sample size 64 (32 per group). Taking into account a potential 20% sample loss, the final sample size reached 40 per group.

The researcher visited the select hospitals to carry out convenience sampling. The participants were then randomly allocated to the intervention and control groups by permuted blocks of four. To prevent selection bias, allocation to the groups was performed by an independent third person and the allocation sequence was concealed in sealed envelopes numbered from 1 to 80. Except for the senior researcher, the other investigators in the clinic were unaware of the size and sequence of the blocks. After the recruitment of the participants, the assigned envelopes were opened based on their order and the subjects were assigned to either the intervention group (A) or the control group (B) [23].

The sampling process was not concurrent in the two select hospitals, and it was first carried out at Allameh Bohlool Gonabadi Hospital and then continued in 17-Shahrivar Hospital.

### Study instruments

Data were collected using several questionnaires, including a demographic and obstetric questionnaire, a labor checklist and the visual analogue scale (VAS) to assess the afterpain intensity.

The instruments are described in detail in the protocol article of this study [23]. The items in the demographic and obstetric questionnaire included the parturient and her husband’s age and education, weight, and obstetric history, i.e., gravidity, parity, postpartum bleeding, dysmenorrhea, gestational age, bleeding in pregnancy, and the position of the placenta.

The labor stage checklist included items such as vital signs in different labor stages, medications used to relieve labor pain or accelerate labor, the duration of each labor stage, neonatal weight, placental weight, and analgesics used in the fourth stage of labor.

VAS is a standard tool with a confirmed validity as per previous studies, including the one by Gallagher (2002) [25]. The reliability of VAS has also been confirmed in various studies, such as the one by Khalilian Muvahhed et al. (2012), which reported the equivalent forms reliability as r = 0.91 [26]. It ranges from 0 to 10, with a higher score indicating the more pain intensity.

### Interventions and outcomes

Before sampling, the researcher’s skill in performing foot reflexology techniques based on the standard protocol was approved by a traditional Chinese medicine and acupuncture specialist after passing several training sessions. At the beginning of the study, the demographic and obstetric questionnaire was completed for the mother by asking questions from her and checking her hospital records. The researcher was present at the mother’s bedside during the second stage and monitored the entire second and third stages of labor and recorded the necessary information in the labor checklist. The participants were blinded to their group allocation (single-blind design). After the third stage of labor, the researcher started the intervention in the experimental group. The following measures were taken to ensure similar conditions were observed for the intervention in both arms; for instance, the room had to be private and a pillow was supposed to be placed under the foot to provide comfort. The same researcher (i.e., the first author) carried out the intervention in both arms. The reflexology group received 4 min of general massage on each foot, followed by 2 min of specific reflexology on each point of the uterus, pituitary, and solar plexus in the form of rotational pressures. Prior to this study, no research had been conducted on the afterpains of vaginal delivery. These points were selected for the reflexology because they have been effective in reducing maternal pain and anxiety during childbirth according to various studies [19]. In a study by Azizi et al., reflexology reduced cesarean section pain [24]. Reflexology was also performed in the second hour postpartum.

The control group received general massage on each foot for 4 min, followed by rotational pressures on a neutral point on the lateral side of the heel (placebo point) for 6 minutes. This step was repeated in the second hour postpartum. The VAS was marked by the participants at each time point of one, two, three and 4 h postpartum in both groups to check their afterpain intensity. Postpartum care was provided to both groups according to the national protocol. The use of NSAIDs, including mefenamic acid, in the third and fourth hours after delivery was also recorded.

### Statistical analysis

SPSS-16 package was used for the statistical analysis of the data. Shapiro-Wilk’s test was used to check the normal distribution of the quantitative variables. The mean scores of the quantitative variables were compared using the independent-samples t-test and Mann-Whitney’s U-test. The qualitative and categorical variables were compared using the Chi-squared test. Friedman’s test was used to evaluate changes in the median score of postpartum pain separately in each group. To compare the postpartum pain scores at the different time points, since the two groups did not match in terms of mefenamic acid dosage in the third and fourth hours, the analysis of covariance (ANCOVA) was used to adjust the effect of mefenamic acid use as a confounder. The level of statistical significance was 0.05.

## Results

**Fig. 1:**
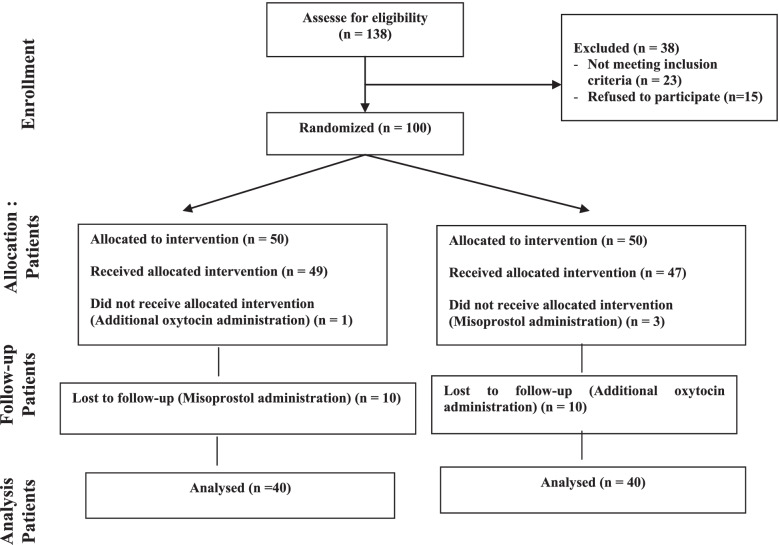
The flow chart of randomized clinical trials on the effect of foot reflexology in the fourth stage of labor on uterine afterpains

This study assessed the eligibility of 138 individuals and included 100 individuals in the study, who were allocated to the two groups (reflexology and control groups). During the study, 20 participants were excluded due to receiving misoprostol or extra oxytocin or showing lack of cooperation. Finally, 80 individuals (40 in the reflexology group and 40 in the control group) were evaluated (Fig. 1).

There was no statistically significant difference between the two groups in terms of demographic characteristics such as maternal age, husband’s age, maternal education, husband’s education, maternal occupation, husband’s occupation, maternal weight, and history of dysmenorrhea (Table 1).

**Table 1:** Baseline sociodemographic characteristics of foot reflexology and control groups

Variable	Foot reflexology group (***n*** = 40)	Control group (***n*** = 40)	***p***-value
Women age (mean ±SD*)*	29.18 ± 4.93	29.10 ± 3.62	0.653^*^
Men age (mean ±SD)	33.67 ± 4.31	33.05 ± 4.11	0.395*
Women education (mean ±SD)	10.82 ± 3.35	9.58 ± 3.52	0.098*
Men education (mean ±SD)	9.70 ± 3.79	10.25 ± 3.71	0.595*
Women job, count (%)			0.210^+^
Housewife	39 (97.50%)	39 (97.50%)	
Employee	1 (2.50%)	1 (2.50%)	
Men job, count (%)			0.714^+^
Manual worker	17 (42.5%)	16 (40%)	
Retired, Employee	6 (15%)	4 (10%)	
Farmer, Self-employed	17 (42.5)	20 (50%)	
Women weight (mean ±SD)	71.30 ± 7.81	75.15 ± 1.02	0.201^*^
Dysmenorrhea, count (%)	20 (50.0%)	21 (52. 5%)	0.823^+^

* Mann-Whitney test was used

+ Chi-square test was used

The dose of mefenamic acid consumed at 4 h postpartum was not significantly different between two groups. Nonetheless, its dose used in the third hour and the total dose of mefenamic acid were significantly higher in the control group (Table 2).

**Table 2:** Mefenamic acid usage in foot reflexology and control groups

Variable	Foot reflexology group (***n*** = 40)	Control group (***n*** = 40)	***p***-value*
Mefenamic acid used in the third hour (mg)	18.7 ± 66.6	75.0 ± 116.0	0.010
Mefenamic acid used in the fourth hour (mg)	6.2 ± 39.5	25.0 ± 75.9	0.170
Total mefenamic acid used (mg)	25.0 ± 75.9	100.0 ± 124.0	0.002

* Mann-Whitney test was used

The mean scores of afterpains were higher in the reflexology group than the control group in the first hour (*P* = 0.05) and second hour (*P* = 0.27) postpartum, but this difference was not statistically significant (Table 3). After adjustment for mefenamic acid usage, the mean scores of afterpains were significantly lower in the reflexology group at3 h (*P* < 0.001) and 4 h (P < 0.001) postpartum. Furthermore, the total mean score of afterpains throughout the 4 h postpartum was significantly lower in the reflexology group (*P* = 0.001) (Table 3). As an additional step of analysis, members of both groups who had not used any painkillers at all (*n* = 60) were also compared on the basis of their group, and the results were found to be the same in both groups (data not shown).

**Table 3:** Comparison of postpartum pain in the first to fourth hour after delivery in foot reflexology and control groups

Variable	Foot reflexology group (***n*** = 40)		Control group (***n*** = 40)		***p***-value^*^
	Mean ± SD	Adjusted Mean ± SE	Mean ± SD	Adjusted Mean ± SE	
Postpartum pain in the first hour	2.82 ± 1.82	–	2.05 ± 1.70	–	0.05*
Postpartum pain in the second hours	2.15 ± 2.00	–	2.55 ± 1.10	–	0.27*
Postpartum pain in the third hours	1.38 ± 1.67	1.69 ± 0.22	3.00 ± 1.06	2.95 ± 0.21	< 0.001#
Postpartum pain in the fourth hours	0.65 ± 1.25	0.7 ± 0.20	2.58 ± 1.35	2.50 ± 0.20	< 0.001†
Sum scores of postpartum pain in the four hours	7.00± 5.38	8.04 ± 0.75	10.17 ± 3.24	10.20 ± 0.70	0.001‡

SD: standard deviation, SE: standard error

*Independent sample t test was used

# Analysis of Covariance (ANCOVA) adjusted for dose of mefenamic acid use at 3rd hour

† Analysis of Covariance (ANCOVA) adjusted for dose of mefenamic acid use at 4rd hour

‡ Analysis of Covariance (ANCOVA) adjusted for total dose of mefenamic acid

**Fig. 2:**
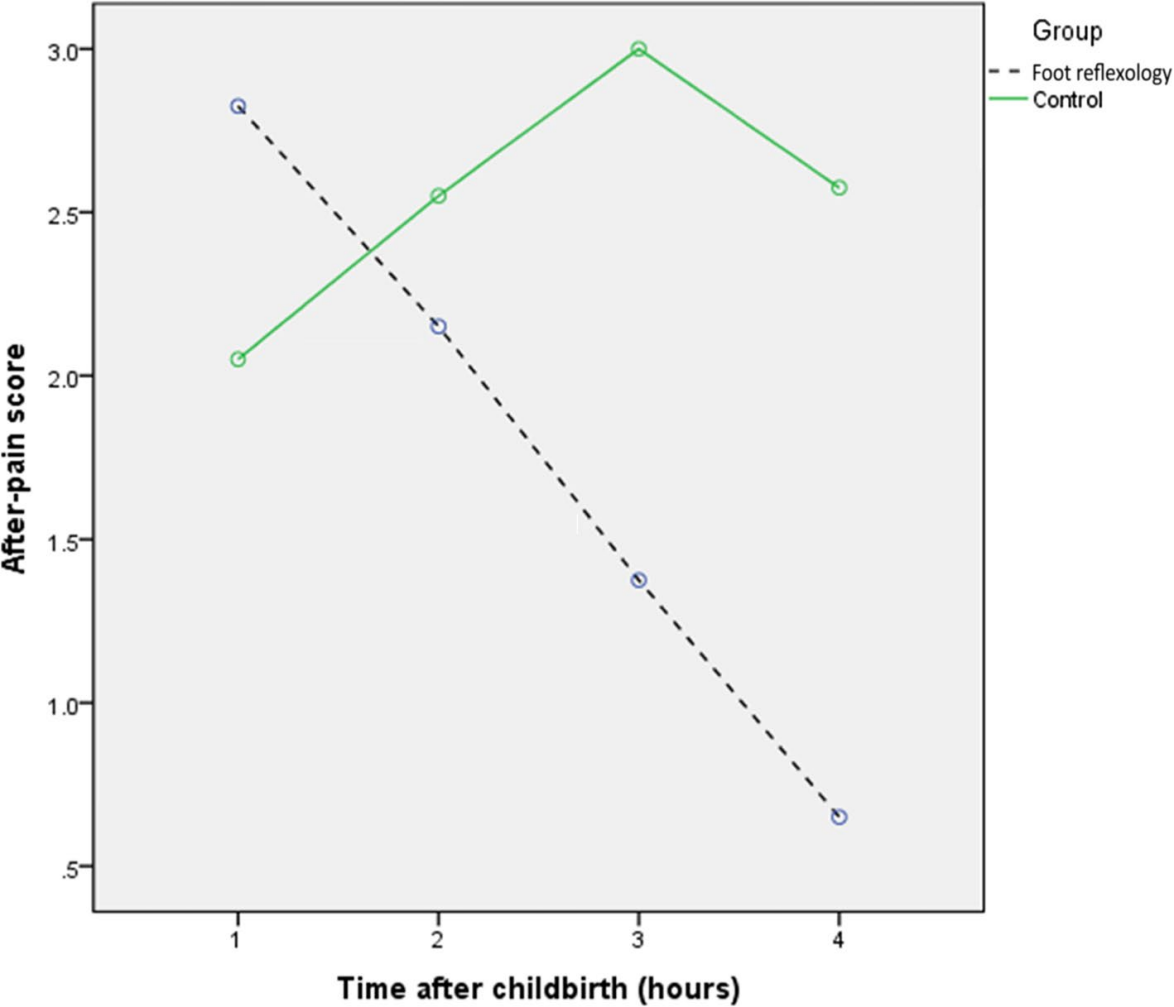
Comparison of the mean postpartum pain in the foot reflexology and control groups

According to Friedman’s test, the median score of afterpains did not change significantly from the first to the fourth hours postpartum in the control group (*P* = 0.056); however, the median score of total afterpains decreased in the reflexology group from the first to fourth hours postpartum (*P* < 0.001) (Fig. 2).

## Discussion

Afterpains felt in the lower abdomen or lower back are one of the most prevalent complaints after labor [27]. NSAIDs are the most common treatment for afterpains [28]. In most studies, however, mefenamic acid has been compared to herbal pain killers to examine the possible side effects of this NSAID. Chananeh et al. (2018) reported that the combination of *Nigella sativa* and mefenamic acid is more effective in reducing afterpains than only mefenamic acid [2]. Abedian et al. (2016) also reported that Chamomile matricaria capsules are more effective in reducing afterpain than mefenamic acid [29]. Since mefenamic acid consumption was effective in reducing afterpain in the cited studies, this interfering variable was controlled in the present study using the ANCOVA.

Complementary medicine used for the treatment of afterpains includes a variety of anti-inflammatory and pain-relieving herbs, such as dill and *Nigella sativa*, or acupressure and acupuncture, which affect the body’s energy channels. Since no study was found on the effect of foot reflexology in the fourth stage of labor on afterpains in our review of literature, the results of the present study are discussed against and compared with the findings of the most relevant articles on the effect of complementary medicine, such as acupressure or acupuncture, on afterpains and the use of foot reflexology in the treatment of pain. This result is consistent with the present findings. The similarity of the results of the two studies can be attributed to the fact that the mechanisms of pain are somewhat similar in labor and afterpains. Ghasemali et al. (2014), Mathew et al. (2016) and Jenabi et al. (2011) reported positive effects for foot reflexology on pain relief in the first stage of labor [30–32]. First, the instruments used and the types of intervention given in the cited studies are similar to those in the present study; second, the mechanism of pain in labor and postpartum pain are somewhat similar; the similarity in the results obtained in the studies can thus be justified.

Mokhtari et al. (2010), Abbaspoor et al. (2014), and Razmjo et al. (2012) reported a reduction in pain after cesarean section using foot reflexology [24, 33, 34]. Their results are consistent with the present findings too. Although part of the pain after cesarean section is related to the incision site, the pain experienced after c-section and vaginal delivery afterpains is generally similar in nature and caused by uterine contractions. Therefore, the pain relief mechanism in the cited studies appears to be similar to that in the present study and is based on foot reflexology massage, which stimulates and activates the neural pathways and the subtle energy pathways associated with the sole and can reduce pain intensity in patients. During massage, blood circulation is improved and the transmission of pain signals through the sensory nerves is inhibited, and pain relief is thus achieved by the release of endorphins and enkephalins [35].

Soltani et al. (2017) used acupressure but found that it did not reduce afterpains in the first and second hours postpartum [36]. This failure to reduce afterpains in the first hour postpartum is consistent with the present study, but the failure observed in the second hour postpartum is not. This difference can be attributed to the place and time of the intervention and also to the fact that the time of pain intensity measurement and participants’ gravidity and parity differ in the two studies; in Soltani’s study, the pain intensity of women with a parity of five or less was measured in one and 2 h postpartum, but in the present study, the severity of pain was measured in women with a parity of two or three up to 4 h postpartum.

In the study by Bakhtyari Nia et al. (2019), the participants were given 10 min of massage on each foot (general foot massage, followed by the application of pressure on the pituitary points, the solar plexus points, the inner arch of the foot and the uterine point) and the severity of postoperative pain was measured in them immediately, 30, 60, 120 min and 6 h after the intervention. In their study, the afterpain experienced in the intervention group was reduced compared to the control group on all the measurement occasions [37]. In the present study, afterpain was measured one, two, three and 4 h after the intervention, and a reduction was noticed in it only at three and 4 h postpartum in the reflexology group. Bakhtyari Nia’s results are consistent with the present findings.

The limitations of the present study include the failure to confirm the accuracy of participants’ statements due to ethical constraints and the influence of personal and genetic characteristics on the pain tolerance. The massage was given by a researcher trained by a complementary medicine specialist. Another limitation was the impossibility of blinding the professionals who performed the reflexology massage.

The strengths of this study include its RCT design, the significance of the subject matter, which has been neglected in literature for the most part, and the use of complementary therapies in the puerperium.

## Conclusion

The results of the present study showed that foot reflexology in the fourth stage of labor has positive effects on and can relieve afterpains. Therefore, it is recommended that foot reflexology be used in clinics for postpartum care as a modality without side effects to improve midwifery services.

## Data Availability

The datasets used and/or analyzed during the present study are available from the corresponding author on reasonable request.

## Abbreviations

RCT: Randomized controlled trial
VAS: Visual analogue scale
IRCT: Iranian Registry of Clinical Trials
